# Supplementary material for: Sexual and reproductive health outcomes of women who experienced violence in Germany: Analysis of the German health interview and examination survey for adults (DEGS1)
Source: PLoS One. 2024 Aug 9;19(8):e0305992. doi: 10.1371/journal.pone.0305992 (PMC11315302; doi:10.1371/journal.pone.0305992)
Supplement: S1 File. S1 Table — S2 Table. Violence victimization in the past 12 months and socio-demographic characteristics. S3 Table. Violence victimization since the age of 16 and socio-demographic characteristics. S4 Table. Childhood violence by parent/caregiver and socio-demographic characteristics. S5 Table. Childhood violence by peers and socio-demographic characteristics. S6 Table. Contraceptive use and socio-demographic characteristics. S7 Table. Sexual and reproductive health and socio-demographic characteristics. S8 Table. Confounders of the association between violence and birth control pill (ever). S9 Table. Confounders of the association between violence and current contraceptive use. S10 Table. Confounders of the association between violence and miscarriage or stillbirth (ever). S11 Table. Confounders of the association between violence and abortion (ever). S12 Table. Comparison between participants with full valid information and those with at least one missing value in one of the violence variables analysed. (DOCX) [file pone.0305992.s001.docx]

**Supplementary material**

**Table S1. DEGS1 translated questions on violence and sexual and reproductive outcomes (own translation)**

| Type of violence | Question | Answers |
| --- | --- | --- |
| *12-months prevalence of physical violence* | In the past 12 months, have you experienced someone physically assaulting you (for example, hitting, slapping, pulling your hair, kicking, threatening you with a weapon or object)?“ | Yes // No |
| *12-months prevalence of psychological violence* | In the last 12 months, have you been belittled by any person (e.g., about the way you look, the way you dress, the way you think, act, or work, or possible disabilities)? Or have you been insulted, called names, threatened, bullied, or pressured? | Yes // No |
| *Lifetime prevalence of violence* | Have you experienced one or more of the acts described above previously in your adult life (since age 16)? | Yes, physical assault // Yes, psychologically impairing acts or threats // No, none of the above |
| *Childhood violence* | Did you experience one or more of the following acts in your childhood and adolescence before the age of 16? Please indicate everything that applies to you, even if it was only once or rarely or not so bad for you: Physical assault by parents/guardians // Physical assault by peers or older youth // Psychological aggression or threats by parents/guardians // Psychological aggression or threats by peers or older adolescents. | Frequently // Occasionally // Rarely // Never |
| SRH outcome |  |  |
| Induced abortion & miscarriage or stillbirth | Please tell us how many live births, miscarriages, stillbirths, and abortions you have had. | Text field: Number of Live births __ // Number of miscarriages or stillbirths __ // number of abortions __ |
| Contraceptive use | Have you ever taken the birth control pill? | Yes // No |
|  | Are you currently using contraceptives? | Yes // No |

**Table S2. Violence victimization in the past 12 months and socio-demographic characteristics**

|  | **Violence in the past 12 months** | | | | | | | |
| --- | --- | --- | --- | --- | --- | --- | --- | --- |
|  | **Physical violence, n= 78 (2.5)** | | | | **Psychological violence, n= 572 (18.2)** | | | |
| **Characteristics** | **n, yes** | **Weighted* % (95% CI)** | **χ²** | **Weighted* OR (95% CI)** | **n, yes** | **Weighted* % (95% CI)** | **χ²** | **Weighted* OR (95% CI)** |
| **Age** |  |  | **.000** |  |  |  | **.000** |  |
| 18-24 years | 26 | 9.3 (6.1 - 13.9) |  | 1 | 95 | 31.7 (26.6 - 37.4) |  | 1 |
| 25-34 years | 22 | 4.7 (2.9 - 7.4) |  | **.281 (.239 - .969)** | 117 | 24.8 (20.5 - 20.6) |  | .708 (.498 - 1.005) |
| 35-44 years | 11 | 2.1 (1.1 - 3.9) |  | **.206 (.094 - .451)** | 123 | 19.5 (16.0 - 23.7) |  | **.522 (.363 - .751)** |
| 45-54 years | 12 | 2.1 (1.0 - 4.2) |  | **.206 (.085 - .500)** | 149 | 17.7 (15.0 - 20.7) |  | **.461 (.341 - .623)** |
| 55-64 years | 7 | 1.0 (0.4 - 2.5) |  | **.100 (.036 - .282)** | 88 | 12.0 (9.5 - 15.1) |  | **.294 (.203 - .425)** |
| **Marital Status** |  |  | **.000** |  |  |  | **.000** |  |
| married | 23 | 1.4 (0.8 - 2.4) |  | 1 | 275 | 14.9 (13.1 - 17.0) |  | 1 |
| married but separated | 6 | 12.6 (5.0 - 28.2) |  | **10.087 (3.190 - 31.895)** | 20 | 37.5 (22.5 - 55.4) |  | **3.422 (1.579 - 7.418)** |
| single | 37 | 5.8 (4.0 - 8.1) |  | **4.287 (2.244 - 8.190)** | 199 | 28.5 (25.0 - 32.2) |  | **2.271 (1.803 - 2.861)** |
| divorced or widowed | 10 | 4.4 (2.2 - 8.7) |  | **3.240 (1.316 - 7.975)** | 70 | 21.0 (16.0 - 27.0) |  | **1.515 (1.070 - 2.145)** |
| **Socioeconomic Status** |  |  | **.002** |  |  |  | **.016** |  |
| low | 19 | 6.3 (3.9 - 19.2) |  | **4.079 (1.797 - 9.257)** | 101 | 25.5 (20.9 - 30.8) |  | **1.638 (1.119 - 2.398)** |
| medium | 44 | 3.0 (2.1 - 4.1) |  | 1.834 (.883 - 3.809) | 341 | 19.3 (17.2 - 21.6) |  | 1.143 (.854 - 1.528) |
| high | 14 | 1.6 (0.9 - 3.0) |  | 1 | 127 | 17.3 (14.0 - 21.1) |  | 1 |
| **Overall health status** |  |  | .366 |  |  |  | **.034** |  |
| very good/good | 65 | 3.5 (2.7 - 4.6) |  | 1 | 419 | 19.1 (17.2 - 21.1) |  | 1 |
| medium/bad/very bad | 13 | 2.5 (1.3 - 4.8) |  | .712 (.338 - 1.497) | 150 | 24.2 (20.1 - 28.8) |  | **1.355 (1.023 - 1.794)** |
| **Social Support** |  |  | .469 |  |  |  | **.000** |  |
| low | 12 | 3.7 (1.8 - 7.5) |  | 1.399 (.581 - 3.368) | 94 | 32.5 (26.2 - 39.5) |  | **2.532 (1.713 - 3.743)** |
| medium | 39 | 3.7 (2.6 - 5.3) |  | 1.401 (.792 - 2.476) | 276 | 20.7 (18.1 - 23.5) |  | **1.369 (1.044 - 1.795)** |
| high | 26 | 2.7 (1.8 - 4.0) |  | 1 | 197 | 16.0 (13.5 - 28.9) |  | 1 |
| **Alcohol consumption** |  |  | .167 |  |  |  | .361 |  |
| never | 11 | 3.4 (1.6 - 7.0) |  | 1 | 76 | 17.8 (13.7 - 22.7) |  | 1 |
| moderate | 39 | 2.6 (1.9 - 3.7) |  | .765 (.337 - 1.739) | 322 | 19.7 (17.4 - 22.2) |  | 1.134 (.795 - 1.617) |
| risk consumption | 27 | 4.7 (3.0 - 7.1) |  | 1.382 (.566 - 3.373) | 163 | 22.0 (18.5 - 25.9) |  | 1.301 (.897 - 1.888) |
| **Number of children** |  |  | **.000** |  |  |  | **.000** |  |
| 0 | 43 | 5.9 (4.3 - 8.2) |  | 1 | 205 | 25.8 (22.5 - 29.5) |  | 1 |
| 1 | 16 | 3.3 (1.9 - 5.7) |  | .539 (.269 - 1.080) | 122 | 21.5 (17.5 - 26.1) |  | .786 (.564 - 1.096) |
| 2 | 12 | 1.5 (0.8 - 3.0) |  | **.244 (.114 - .522)** | 154 | 16.3 (13.7 - 19.4) |  | **.560 (.428 - 7.34)** |
| 3+ | 4 | 1.4 (0.5 - 3.8) |  | **.229 (.077 - .686)** | 60 | 12.9 (9.6 - 17.3) |  | **.427 (.290 - .628)** |
| **Chronic conditions** |  |  | .077 |  |  |  | .575 |  |
| **any** | 17 | 2.1 (1.2 - 2.6) |  | .577 (.312 - 1.069) | 157 | 20.5 (17.1 - 24.4) |  | 1.076 (.832 - 1.392) |
| non | 56 | 3.5 (2.7 - 4.6) |  | 1 | 374 | 19.4 (17.4 - 21.5) |  | 1 |

* Population weights according to [33]; 95%CI= 95% Confidence Interval; OR= Odds ratio; 1= Reference group

**Table S3. Violence victimization since the age of 16 and socio-demographic characteristics**

|  | **Violence since the age of 16** | | | | | | | |
| --- | --- | --- | --- | --- | --- | --- | --- | --- |
|  | **Physical violence, n= 264 (8.3%)** | | | | **Psychological violence, n= 628 (19.9%)** | | | |
| **Characteristics** | **n, yes** | **Weighted* % (95% CI)** | **χ²** | **Weighted* OR (95% CI)** | **n, yes** | **Weighted* % (95% CI)** | **χ²** | **Weighted* OR (95% CI)** |
| **Age** |  |  | .425 |  |  |  | **.000** |  |
| 18-24 years | 33 | 9.7 (6.8 - 13.7) |  | 1 | 90 | 29.0 (24.0 - 34.7) |  | 1 |
| 25-34 years | 47 | 10.4 (7.6 - 14.0) |  | 1.085 (.637 - 1.848) | 135 | 28.7 (23.6 - 34.4) |  | .983 (.673 - 1.437) |
| 35-44 years | 59 | 11.4 (8.3 - 15.6) |  | 1.203 (.715 – 2.026) | 128 | 21.6 (17.5 - 26.3) |  | **.674 (.463 - .979)** |
| 45-54 years | 68 | 8.6 (6.5 - 11.3) |  | .877 (.527 - 1.459) | 182 | 21.2 (18.4 - 24.5) |  | **.659 (.484 - .898)** |
| 55-64 years | 57 | 7.8 (5.6 - 10.7) |  | .788 (.457 - 1.358) | 93 | 13.1 (10.6 - 16.2) |  | **.370 (.257 - .533)** |
| **Marital Status** |  |  | **.000** |  |  |  | **.000** |  |
| married | 118 | 6.8 (5.6 - 8.2) |  | 1 | 289 | 16.5 (14.7 - 18.5) |  | 1 |
| married but separated | 6 | 13.8 (5.3 - 31.5) |  | 2.200 (.739 - 6.551) | 20 | 45.8 (30.3 - 62.2) |  | **4.281 (2.190 - 8.370)** |
| single | 78 | 10.4 (8.1 - 13.3) |  | **1.593 (1.116 - 2.274)** | 215 | 28.8 (25.2 - 32.7) |  | **2.048 (1.623 - 2.586)** |
| divorced or widowed | 55 | 17.9 (13.2 - 23.7) |  | **2.993 (1.965 - 4.557)** | 95 | 28.2 (22.2 - 35.0) |  | **1.985 (1.415 - 2.783)** |
| **Socioeconomic Status** |  |  | .101 |  |  |  | .558 |  |
| low | 47 | 12.2 (9.0 - 16.5) |  | **1.669 (1.020 - 2.731)** | 84 | 23.4 (18.2 - 29.6) |  | .988 (.656 - 1.487) |
| medium | 159 | 9.0 (7.5 - 10.9) |  | 1.188 (.793 - 1.780) | 381 | 21.2 (19.0 - 23.6) |  | .870 (.654 - 1.156) |
| high | 55 | 7.7 (5.7 - 10.4) |  | 1 | 160 | 23.6 (19.6 - 28.2) |  | 1 |
| **Overall health status** |  |  | .062 |  |  |  | **.001** |  |
| very good/good | 185 | 8.8 (7.4 - 10.4) |  | 1 | 455 | 20.4 (18.5 - 22.4) |  | 1 |
| medium/bad/very bad | 76 | 11.9 (9.1 - 15.4) |  | 1.397 (.982 - 1.988) | 169 | 28.9 (23.9 - 34.4) |  | **1.583 (1.201 - 2.087)** |
| **Social Support** |  |  | **.002** |  |  |  | **.002** |  |
| low | 46 | 16.9 (12.1 - 23.0) |  | **2.248 (1.402 - 3.605)** | 80 | 29.1 (21.6 - 38.0) |  | **1.882 (1.215 - 2.915)** |
| medium | 114 | 9.0 (7.2 - 11.1) |  | 1.091 (.769 - 1.548) | 330 | 24.5 (21.9 - 27.2) |  | **1.483 (1.152 - 1.909)** |
| high | 101 | 8.3 (6.5 - 10.4) |  | 1 | 215 | 17.9 (15.2 - 21.0) |  | 1 |
| **Alcohol consumption** |  |  | **.000** |  |  |  | .131 |  |
| never | 38 | 8.5 (5.9 - 12.1) |  | 1 | 81 | 18.0 (14.2 - 22.6) |  | 1 |
| moderate | 134 | 7.7 (6.4 - 9.2) |  | .898 (.566 - 1.424) | 356 | 22.0 (19.6 - 24.6) |  | 1.281 (.921 - 1.783) |
| risk consumption | 90 | 14.0 (10.8 - 18.1) |  | **1.764 (1.075 - 2.893)** | 180 | 24.4 (20.5 - 28.9) |  | **1.472 (1.026 - 2.112)** |
| **Number of children** |  |  | .472 |  |  |  | **.000** |  |
| 0 | 75 | 9.4 (7.4 - 11.9) |  | 1 | 234 | 28.6 (24.9 - 32.5) |  | 1 |
| 1 | 71 | 11.5 (8.7 - 15.3) |  | 1.259 (.837 - 1.894) | 138 | 22.6 (18.7 - 27.2) |  | **.731 (.538 - .992)** |
| 2 | 75 | 8.5 (6.4 - 11.2) |  | .894 (.596 - 1.341) | 160 | 18.1 (14.9 - 21.7) |  | **.551 (.408 - .745)** |
| 3+ | 32 | 9.6 (6.4 - 14.2) |  | 1.025 (.624 - 1.684) | 66 | 16.7 (12.5 - 22.0) |  | **.502 (.342 - .736)** |
| **Chronic conditions** |  |  | .051 |  |  |  | **.005** |  |
| **any** | 87 | 11.6 (9.1 - 14.7) |  | 1.396 (.997 - 1.956) | 192 | 25.8 (22.3 - 29.7) |  | **1.380 (1.105 - 1.723)** |
| non | 159 | 8.6 (7.2 - 10.2) |  | 1 | 396 | 20.2 (18.1 - 22.4) |  | 1 |

* Population weights according to [33]; 95%CI= 95% Confidence Interval; OR= Odds ratio; 1= Reference group

**Table S4. Childhood violence by parent/caregiver and socio-demographic characteristics**

|  | **Childhood violence by parent/caregiver** | | | | | | | |
| --- | --- | --- | --- | --- | --- | --- | --- | --- |
|  | **Physical violence, n= 1,078 (34.2)** | | | | **Psychological violence, n= 730 (23.2)** | | | |
| **Characteristics** | **n, yes** | **Weighted* % (95% CI)** | **χ²** | **Weighted* OR (95% CI)** | **n, yes** | **Weighted* % (95% CI)** | **χ²** | **Weighted* OR (95% CI)** |
| **Age** |  |  | **.000** |  |  |  | .378 |  |
| 18-24 years | 85 | 27.7 (22.7 - 33.2) |  | 1 | 76 | 26.2 (21.4 - 31.6) |  | 1 |
| 25-34 years | 124 | 25.6 (21.4 - 30.3) |  | .901 (.643 - 1.262) | 118 | 26.7 (21.6 - 32.5) |  | 1.028 (.696 - 1.519) |
| 35-44 years | 247 | 40.9 (36.2 - 45.7) |  | **1.807 (1.328 - 2.459)** | 164 | 27.0 (23.1 - 31.3) |  | 1.042 (.752 - 1.445) |
| 45-54 years | 341 | 40.7 (36.8 - 44.7) |  | **1.794 (1.302 - 2.472)** | 221 | 27.3 (23.7 - 31.3) |  | 1.060 (.764 - 1.472) |
| 55-64 years | 281 | 40.0 (34.8 - 45.3) |  | **1.743 (1.231 - 2.467)** | 151 | 21.7 (17.9 - 26.2) |  | .784 (.548 - 1.119) |
| **Marital Status** |  |  | **.000** |  |  |  | **.009** |  |
| married | 668 | 37.4 (34.6 - 40.3) |  | 1 | 405 | 23.3 (21.0 - 25.9) |  | 1 |
| married but separated | 28 | 44.7 (29.4 - 61.1) |  | 1.355 (.687 - 2.670) | 19 | 32.4 (19.6 - 48.5) |  | 1.575 (.810 - 3.064) |
| single | 218 | 27.8 (24.1 - 31.9) |  | **.646 (.513 - .813)** | 196 | 27.4 (23.8 - 31.4) |  | 1.241 (.971 - 1.588) |
| divorced or widowed | 153 | 46.7 (40.5 - 53.0) |  | **1.470 (1.113 - 1.941)** | 103 | 33.7 (27.4 - 40.8) |  | **1.675 (1.218 - 2.302)** |
| **Socioeconomic Status** |  |  | .989 |  |  |  | .659 |  |
| low | 128 | 36.1 (30.7 - 41.8) |  | 1.019 (.755 - 1.376) | 91 | 26.1 (20.9 - 32.0) |  | .920 (.632 - 1.338) |
| medium | 661 | 36.0 (33.3 - 38.8) |  | 1.015 (.815 - 1.266) | 440 | 25.3 (22.6 - 28.1) |  | .882 (.693 - 1.123) |
| high | 285 | 35.7 (31.4 - 40.1) |  | 1 | 197 | 27.7 (23.9 - 31.9) |  | 1 |
| **Overall health status** |  |  | **.000** |  |  |  | **.000** |  |
| very good/good | 807 | 33.4 (31.1 - 35.8) |  | 1 | 528 | 23.5 (21.3 - 25.9) |  | 1 |
| medium/bad/very bad | 266 | 45.0 (39.7 - 50.3) |  | **1.626 (1.285 - 2.057)** | 198 | 34.2 (29.4 - 39.4) |  | **1.689 (1.300 - 2.195)** |
| **Social Support** |  |  | **.020** |  |  |  | **.000** |  |
| low | 130 | 44.5 (37.0 - 52.3) |  | **1.620 (1.135 - 2.312)** | 103 | 39.0 (31.3 - 47.2) |  | **2.377 (1.621 - 3.484)** |
| medium | 513 | 36.5 (33.4 - 39.8) |  | 1.161 (.944 - 1.427) | 360 | 27.3 (24.7 - 30.1) |  | **1.399 (1.135 - 1.723)** |
| high | 427 | 33.1 (29.8 - 36.6) |  | 1 | 263 | 21.2 (18.5 - 24.2) |  | 1 |
| **Alcohol consumption** |  |  | .488 |  |  |  | .065 |  |
| never | 138 | 35.2 (29.2 - 41.6) |  | 1 | 95 | 23.9 (19.3 - 29.2) |  | 1 |
| moderate | 608 | 34.9 (32.2 - 37.7) |  | .987 (.715 - 1.362) | 405 | 24.7 (22.1 - 27.4) |  | 1.043 (.759 - 1.432) |
| risk consumption | 310 | 38.0 (33.6 - 42.7) |  | 1.133 (.810 - 1.585) | 218 | 29.7 (25.9 - 33.9) |  | 1.347 (.967 - 1.877) |
| **Number of children** |  |  | **.007** |  |  |  | .829 |  |
| 0 | 259 | 30.5 (26.8 - 34.5) |  | 1 | 213 | 27.0 (23.5 - 30.8) |  | 1 |
| 1 | 237 | 36.6 (32.1 - 41.3) |  | **1.314 (1.003 - 1.723)** | 156 | 24.9 (20.8 - 29.5) |  | .897 (.672 - 1.198) |
| 2 | 371 | 37.4 (33.4 - 41.5) |  | **1.360 (1.062 - 1.743)** | 220 | 24.8 (21.2 - 28.8) |  | .893 (.683 - 1.168) |
| 3+ | 160 | 42.7 (36.1 - 49.6) |  | **1.699 (1.227 - 2.351)** | 104 | 25.6 (20.7 - 31.3) |  | .932 (.662 - 1.312) |
| **Chronic conditions** |  |  | **.000** |  |  |  | **.000** |  |
| **any** | 338 | 44.7 (40.3 - 49.3) |  | **1.648 (1.339 - 2.027)** | 233 | 32.1 (27.8 - 36.8) |  | **1.558 (1.242 - 1.955)** |
| non | 693 | 32.9 (30.5 - 35.5) |  | 1 | 459 | 23.3 (21.2 - 25.5) |  | 1 |

* Population weights according to [33]; 95%CI= 95% Confidence Interval; OR= Odds ratio; 1= Reference group

**Table S5. Childhood violence by peers and socio-demographic characteristics**

|  | **Childhood violence by peers** | | | | | | | |
| --- | --- | --- | --- | --- | --- | --- | --- | --- |
|  | **Physical violence, n= 916 (29.1)** | | | | **Psychological violence, n= 847 (26.9)** | | | |
| **Characteristics** | **n, yes** | **Weighted* % (95% CI)** | **χ²** | **Weighted* OR (95% CI)** | **n, yes** | **Weighted* % (95% CI)** | **χ²** | **Weighted* OR (95% CI)** |
| **Age** |  |  | **.000** |  |  |  | .378 |  |
| 18-24 years | 98 | 32.7 (27.4 - 38.5) |  | 1 | 132 | 44.1 (37.9 - 50.6) |  | 1 |
| 25-34 years | 143 | 30.7 (25.1 - 36.9) |  | .910 (.635 - 1.304) | 167 | 33.8 (28.0 - 40.2) |  | **.646 (.442 - .946)** |
| 35-44 years | 232 | 37.8 (33.5 - 42.4) |  | 1.250 (.920 - 1.699) | 214 | 34.1 (29.7 - 38.8) |  | **.656 (.472 - .910)** |
| 45-54 years | 250 | 29.5 (26.2 - 33.1) |  | .860 (.631 - 1.171) | 218 | 25.6 (22.4 - 29.1) |  | **.436 (.320 - .594)** |
| 55-64 years | 193 | 27.5 (23.1 - 32.4) |  | .780 (.551 - 1.104) | 116 | 17.2 (14.0 - 21.0) |  | **.264 (.185 - .376)** |
| **Marital Status** |  |  | .069 |  |  |  | **.000** |  |
| married | 534 | 30.5(27.9 - 33.3) |  | 1 | 432 | 24.8 (22.5 - 27.2) |  | 1 |
| married but separated | 20 | 26.0 (14.7 - 41.6) |  | .799 (.386 - 1.657) | 21 | 28.0 (16.5 - 43.4) |  | 1.182 (.598 - 2.335) |
| single | 227 | 30.1 (26.2 - 34.4) |  | .983 (.774 - 1.248) | 285 | 39.6 (35.6 - 43.8) |  | **1.995 (1.627 - 2.446)** |
| divorced or widowed | 122 | 39.7 (32.5 - 47.3) |  | **1.498 (1.076 - 2.084)** | 98 | 31.7 (25.1 - 30.2) |  | 1.410 (.996 - 1.997) |
| **Socioeconomic Status** |  |  | .816 |  |  |  | .862 |  |
| low | 132 | 32.6 (27.5 - 38.2) |  | 1.049 (.785 - 1.402) | 118 | 30.2 (25.3 - 35.6) |  | .963 (.719 - 1.289) |
| medium | 548 | 30.9 (28.3 - 33.7) |  | .970 (.779 - 1.209) | 506 | 29.7 (27.0 - 32.5) |  | .937 (.739 - 1.189) |
| high | 230 | 31.6 (27.9 - 35.6) |  | 1 | 221 | 31.0 (27.3 - 35.0) |  | 1 |
| **Overall health status** |  |  | .182 |  |  |  | .606 |  |
| very good/good | 709 | 30.8 (28.6 - 33.1) |  | 1 | 659 | 29.6 (27.4 - 31.9) |  | 1 |
| medium/bad/very bad | 202 | 34.3 (29.5 - 39.5) |  | 1.174 (.927 - 1.488) | 184 | 30.9 (26.4 - 35.9) |  | 1.065 (.837 - 1.354) |
| **Social Support** |  |  | **.004** |  |  |  | **.003** |  |
| low | 108 | 37.3 (30.2 - 45.0) |  | **1.576 (1.103 - 2.251)** | 95 | 36.5 (29.7 - 43.9) |  | **1.629 (1.153 - 2.303)** |
| medium | 473 | 34.0 (31.2 - 36.9) |  | **1.364 (1.108 - 1.678)** | 438 | 32.5 (29.6 - 35.5) |  | **1.361 (1.103 - 1.678)** |
| high | 330 | 27.4 (24.2 - 30.8) |  | 1 | 312 | 26.1 (23.1 - 29.4) |  | 1 |
| **Alcohol consumption** |  |  | **.001** |  |  |  | **.000** |  |
| never | 90 | 25.4 (20.8 - 30.6) |  | 1 | 90 | 21.2 (17.2 - 25.8) |  | 1 |
| moderate | 522 | 30.4 (27.7 - 33.2) |  | 1.283 (.947 - 1.739) | 487 | 30.3 (27.7 - 33.1) |  | **1.622 (1.222 - 2.151)** |
| risk consumption | 289 | 37.0 (33.0 - 41.2) |  | **1.725 (1.285 - 2.316)** | 261 | 35.1 (31.2 - 39.3) |  | **2.015 (1.440 - 2.820)** |
| **Number of children** |  |  | .887 |  |  |  | **.000** |  |
| 0 | 250 | 31.8 (28.3 - 35.4) |  | 1 | 289 | 36.9 (33.0 - 41.0) |  | 1 |
| 1 | 201 | 33.0 (27.8 - 38.5) |  | 1.057 (.791 - 1.413) | 182 | 30.0 (25.1 - 35.4) |  | **.732 (.543 - .987)** |
| 2 | 310 | 31.2 (27.4 - 35.3) |  | .976 (.762 - 1.249( | 244 | 26.7 (23.4 - 30.4) |  | **.624 (.487 - .799)** |
| 3+ | 111 | 29.9 (24.4 - 36.2) |  | .918 (.661 - 1.277) | 91 | 20.6 (16.2 - 25.8) |  | **.444 (.317 - .623)** |
| **Chronic conditions** |  |  |  |  |  |  |  |  |
| **any** | 257 | 35.0 (31.3 - 39.0) | **.021** | **1.266 (1.037 - 1.546)** | 238 | 32.6 (28.7 - 36.7) | .089 | 1.209 (.971 - 1.505) |
| non | 608 | 29.8 (27.4 - 32.3) |  | 1 | 563 | 28.6 (26.1 - 31.1) |  | 1 |

* Population weights according to [33]; 95%CI= 95% Confidence Interval; OR= Odds ratio; 1= Reference group

**Table S6. Contraceptive use and socio-demographic characteristics**

|  | **Contraceptive use** | | | | | | | |
| --- | --- | --- | --- | --- | --- | --- | --- | --- |
|  | **Birth control pill (ever), n= 2,632 (83.6%)** | | | | **Current contraceptive use, n= 1,207 (38.3%)** | | | |
| **Characteristics** | **n, yes** | **Weighted* % (95% CI)** | **χ²** | **Weighted* OR (95% CI)** | **n, yes** | **Weighted* % (95% CI)** | **χ²** | **Weighted* OR (95% CI)** |
| **Age** |  |  | **.000** |  |  |  | **.000** |  |
| 18-24 years | 242 | 73.8 (67.6 - 79.1) |  | 1 | 233 | 71.8 (65.5 - 77.3) |  | 1 |
| 25-34 years | 428 | 87.9 (82.9 - 90.3) |  | **1.381 (1.548 - 3.663)** | 320 | 67.5 (61.9 - 72.7) |  | **.817 (.561 - 1.191)** |
| 35-44 years | 590 | 86.1 (82.4 - 89.1) |  | **2.195 (1.446 - 3.331)** | 385 | 58.8 (54.0 - 63.4) |  | **.561 (.385 - .816)** |
| 45-54 years | 763 | 85.5(82.3 - 88.2) |  | **2.092 (1.476 - 2.965)** | 255 | 29.6 (26.0 - 33.5) |  | **.165 (.116 - .235)** |
| 55-64 years | 609 | 80.1 (76.2 - 83.6) |  | 1.434 (.980 - 2.099) | 14 | 2.2 (1.1 - 4.3) |  | **.009 (.004 - .019)** |
| **Marital Status** |  |  | **.001** |  |  |  | **.000** |  |
| married | 1,647 | 84.9 (82.7 - 86.8) |  | 1 | 642 | 38.0 (35.4 - 40.8) |  | 1 |
| married but separated | 55 | 89.6 (70.2 - 96.9) |  | 1.538 (.408 - 5.805) | 13 | 30.0 (16.5 - 48.3) |  | .699 (.320 - 1.527) |
| single | 612 | 79.8 (76.3 - 83.0) |  | **.705 (.551 - .902)** | 463 | 63.4 (59.2 - 67.4) |  | **2.825 (2.280 - 3.499)** |
| divorced or widowed | 298 | 83.2 (77.7 - 87.6) |  | .884 (.595 - 1.312) | 77 | 23.2 (17.4 - 30.4) |  | **.493 (.330 - .737)** |
| **Socioeconomic Status** |  |  | **.000** |  |  |  | **.003** |  |
| low | 295 | 68.6 (63.2 - 73.5) |  | **.284 (.200 - .405)** | 135 | 35.1 (29.9 - 40.7) |  | **.697 (.518 - .936)** |
| medium | 1,667 | 85.8 (83.7 - 87.8) |  | **.789 (.562 - 1.106)** | 774 | 45.7 (43.1 - 48.3) |  | 1.081 (.878 - 1.331) |
| high | 663 | 88.5 (85.4 - 91.0) |  | 1 | 294 | 43.7 (39.4 - 48.2) |  | 1 |
| **Overall health status** |  |  | **.000** |  |  |  | **.001** |  |
| very good/good | 2,128 | 85.4 (83.5 - 87.1) |  | 1 | 1044 | 47.5 (45.3 - 49.7) |  | 1 |
| medium/bad/very bad | 494 | 76.0 (71.1 - 80.3) |  | **.542 (.403 - .731)** | 161 | 29.4 (25.1 - 34.0) |  | **.697 (.518 - .936)** |
| **Social Support** |  |  | **.000** |  |  |  | .163 |  |
| low | 229 | 72.2 (64.7 - 78.6) |  | **.440 (.291 - .664)** | 86 | 36.7 (29.6 - 44.4) |  | .742 (.522 - 1.054) |
| medium | 1,273 | 83.9 (81.3 - 86.2) |  | .883 (.662 - 1.178) | 598 | 44.3 (41.2 - 47.6) |  | 1.020 (.849 - 1.226) |
| high | 1,116 | 85.5 (82.7 - 87.9) |  | 1 | 515 | 43.8 (40.8 - 46.9) |  | 1 |
| **Alcohol consumption** |  |  | **.000** |  |  |  | **.000** |  |
| never | 284 | 67.0 (61.0 - 72.4) |  | 1 | 101 | 27.6 (21.9 - 34.1) |  | 1 |
| moderate | 1,549 | 85.4 (83.1 - 87.3) |  | **2.874 (2.114 - 3.907)** | 725 | 46.0 (43.5 - 48.6) |  | **2.239 (1.598 - 3.137)** |
| risk consumption | 764 | 88.5 (85.6 - 90.9) |  | **3.807 (2.580 - 5.619)** | 364 | 46.0 (43.5 - 48.6) |  | **2.283 (1.621 - 3.217)** |
| **Number of children** |  |  | **.003** |  |  |  | **.000** |  |
| 0 | 672 | 80.6 (77.4 - 83.5) |  | 1 | 442 | 57.1 (53.1 - 61.1) |  | 1 |
| 1 | 607 | 87.9 (84.0 - 90.9) |  | **1.748 (1.193 - 2.561)** | 238 | 37.7 (32.9 - 42.7) |  | **.454 (.349 - .590)** |
| 2 | 908 | 84.5 (81.5 - 87.1) |  | **1.314 (.979 - 1.763)** | 382 | 42.0 (38.4 - 45.6) |  | **.543 (.431 - .684)** |
| 3+ | 320 | 78.8 (73.6 - 83.2) |  | .894(.646 - 1.238) | 98 | 27.0 (21.5 - 33.3) |  | **.277 (.197 - .391)** |
| **Chronic conditions** |  |  | .354 |  |  |  | **.000** |  |
| **any** | 676 | 82.1 (78.4 - 85.3) | .354 | .876 (.661 - 1.161) | 222 | 29.7 (25.9 - 33.8) |  | **.455 (.370 - .559)** |
| non | 1,841 | 83.9 (81.8 - 85.9) |  | 1 | 934 | 48.1 (45.8 - 50.5) |  | 1 |

* Population weights according to [33]; 95%CI= 95% Confidence Interval; OR= Odds ratio; 1= Reference group

**Table S7. Sexual and reproductive health and socio-demographic characteristics**

|  | **Sexual and reproductive health** | | | | | | | |
| --- | --- | --- | --- | --- | --- | --- | --- | --- |
|  | **Ever miscarriage or stillbirth, n=478 (15.2%)** | | | | **Ever abortion, n=468 (14.9%)** | | | |
| **Characteristics** | **n, yes** | **Weighted* % (95% CI)** | **χ²** | **Weighted* OR (95% CI)** | **n, yes** | **Weighted* % (95% CI)** | **χ²** | **Weighted OR* (95% CI)** |
| **Age** |  |  | **.000** |  |  |  | **.000** |  |
| 18-24 years | 5 | 1.6 (0.6 - 4.2) |  | 1 | 9 | 3.4 (1.7 - 6.7) |  | 1 |
| 25-34 years | 38 | 8.6 (5.9 - 12.5) |  | **5.927 (1.994 - 17.621)** | 35 | 8.2 (5.3 - 12.6) |  | **2.535 (1.165 - 5.514)** |
| 35-44 years | 104 | 20.0 (15.8 - 25.0) |  | **15.685 (5.397 - 45.583)** | 101 | 18.8 (14.6 - 23.9) |  | **6.542 (3.034 - 13.970)** |
| 45-54 years | 188 | 29.8 (25.7 - 34.3) |  | **26.616 (9.484 - 74.690)** | 156 | 21.2 (17.6 - 25.3) |  | **7.600 (3.634 - 15.896)** |
| 55-64 years | 143 | 28.8 (24.0 - 34-1) |  | **25.321 (8.785 - 72.982)** | 167 | 33.2 (28.4 - 38.4) |  | **14.059 (6.522 - 30.306)** |
| **Marital Status** |  |  | **.000** |  |  |  | **.000** |  |
| married | 353 | 25.0 (22.5 - 27.7) |  | 1 | 290 | 19.2 (16.4 - 22.4) |  | 1 |
| married but separated | 12 | 23.8 (12.2 - 41.2) |  | .934 (.408 - 2.136) | 13 | 26.5 (13.8 - 44.8) |  | 1.512 (.661 - 3.457) |
| single | 35 | 4.5 (3.0 - 6.7) |  | **.143 (.092 - .222)** | 62 | 7.8 (5.7 - 10.7) |  | **.356 (.240 - .528)** |
| divorced or widowed | 73 | 27.5 (21.2 - 34.9) |  | 1.138 (.787 - 1.647) | 99 | 34.8 (27.6 - 42.7) |  | **2.239 (1.517 - 3.303)** |
| **Socioeconomic Status** |  |  | .254 |  |  |  | .654 |  |
| low | 69 | 22.4 (16.9 - 28.9) |  | 1.298 (.839 - 2.006) | 57 | 18.9 (14.2 - 24.8) |  | 1.119 (.756 - 1.658) |
| medium | 284 | 17.7 (14.7 - 19.9) |  | .969 (.719 - 1.304) | 296 | 16.6 (14.3 - 19.1) |  | .954 (.709 - 1.283) |
| high | 123 | 18.2 (14.8 - 22.1) |  | 1 | 114 | 17.3 (13.9 - 21.2) |  | 1 |
| **Overall health status** |  |  | **.001** |  |  |  | **.001** |  |
| very good/good | 359 | 16.7 (14.9 - 18.7) |  | 1 | 344 | 15.4 (13.4 - 17.6) |  | 1 |
| medium/bad/very bad | 118 | 26.2 (21.3 - 31.9) |  | **1.772 (1.274 - 2.467)** | 121 | 23.7 (19.1 - 29.1) |  | **1.709 (1.262 - 2.314)** |
| **Social Support** |  |  | .427 |  |  |  | .067 |  |
| low | 45 | 19.1 (13.8 - 25.7) |  | .955 (.636 - 1.434) | 57 | 23.0 (16.2 - 31.6) |  | **1.679 (1.060 - 2.660)** |
| medium | 218 | 17.3 (15.1 - 19.9) |  | .851 (.662 - 1.094) | 245 | 17.6 (15.1 - 20.3) |  | 1.197 (.924 - 1.551) |
| high | 211 | 19.8 (17.1 - 22.7) |  | 1 | 163 | 15.1 (12.6 - 18.0) |  | 1 |
| **Alcohol consumption** |  |  | .164 |  |  |  | .271 |  |
| never | 62 | 18.9 (13.9 - 25.3) |  | 1 | 65 | 19.4 (14.4 - 25.6) |  | 1 |
| moderate | 290 | 19.7 (17.6 - 22.0) |  | 1.050 (.694 - 1.589) | 283 | 17.7 (15.3 - 20.5) |  | .895 (.602 - 1.332) |
| risk consumption | 118 | 15.4 (12.5 - 18.8) |  | .778 (.496 - 1.219) | 115 | 14.8 (11.6 - 18.6) |  | .720 (.459 - 1.128) |
| **Number of children** |  |  | **.000** |  |  |  | **.000** |  |
| 0 | 26 | 2.8 (1.8 - 4.4) |  | 1 | 38 | 4.7 (3.2 - 6.8) |  | 1 |
| 1 | 109 | 21.1 (17.4 - 25.3) |  | **9.158 (5.399 - 15.534)** | 119 | 20.4 (16.3 - 25.1) |  | **5.217 (3.282 - 8.293)** |
| 2 | 213 | 28.3 (24.9 - 32.1) |  | **13.539 (8.091 - 22.656)** | 189 | 23.0 (19.2 - 27.2) |  | **6.086 (3.936 - 9.412)** |
| 3+ | 99 | 34.7 (27.6 - 42.6) |  | **18.187 (10.082 - 32.807)** | 87 | 27.6 (21.4 - 34.8) |  | **7.796 (4.667 - 13.023)** |
| **Chronic conditions** |  |  |  |  |  |  |  |  |
| **any** | 139 | 21.6 (17.9 - 25.8) | .059 | 1.308 (.990 - 1.729) | 150 | 20.4 (16.8 - 24.6) | **.018** | **1.400 (1.058 - 1.852)** |
| non | 321 | 17.4 (15.6 - 19.4) |  | 1 | 291 | 15.5 (13.3 - 17.8) |  | 1 |

* Population weights according to [33]; 95%CI= 95% Confidence Interval; OR= Odds ratio; 1= Reference group

**Table S8. Confounders of the association between violence and birth control pill (ever)**

|  | **Sexual and reproductive health outcome** | | | | | |
| --- | --- | --- | --- | --- | --- | --- |
|  | **Birth control pill (ever)** | | | | | |
| **Violence** | **Crude Model Weighted OR (95% CI)** | **Model 1 Weighted aOR (95% CI)** | **Model 2 Weighted aOR (95% CI)** | **Model 3 Weighted aOR (95% CI)** | **Model 4 Weighted aOR (95% CI)*** | **Model 5 Weighted aOR (95% CI)*** |
| **Physical violence 12 months** |  |  |  |  |  |  |
| no | 1 | 1 | 1 | 1 | 1 | 1 |
| yes | **.469 (.252 - .875)** | .587 (.314 - 1.098) | .567 (.301 - 1.070) | .618 (.301 - 1.269) | .748 (.302 - 1.851) | .753 (.301 - 1.886) |
| **Psychological violence 12 months** |  |  |  |  |  |  |
| no | 1 | 1 | 1 | 1 | 1 | 1 |
| yes | .993 (.731 - 1.350) | 1.100 (.792 - 1.529) | 1.071 (.769 - 1.493) | .999 (.697 - 1.433 | 1.152 (.775 - 1.713) | 1.143 (.768 - 1.702) |
| **Physical violence since the age of 16** |  |  |  |  |  |  |
| no | 1 | 1 | 1 | 1 | 1 | 1 |
| yes | .930 (.598 - 1.446) | 1.014 (.655 - 1.572) | 1.021 (.652 - 1.598) | .867 (.529 - 1.421) | 1.120 (.670 - 1.874) | 1.162 (.684 - 1.975) |
| **Psychological violence since the age of 16** |  |  |  |  |  |  |
| no | 1 | 1 | 1 | 1 | 1 | 1 |
| yes | .981 (.724 - 1.331) | 1.033 (.730 - 1.460) | 1.018 (.709 - 1.462) | .928 (.641 - 1.342) | .967 (.649 - 1.440) | .955 (.643 - 1.419) |
| **Physical violence by parent/caregiver** |  |  |  |  |  |  |
| never | 1 | 1 | 1 | 1 | 1 | 1 |
| ever | **1.338 (1.033 - 1.735)** | **1.400 (1.070 - 1.831)** | **1.400 (1.063 - 1.843)** | **1.356 (1.015 - 1.810)** | 1.347 (.966 - 1.877) | 1.376 (.984 - 1.925) |
| **Psychological violence by parents/caregiver** |  |  |  |  |  |  |
| never | 1 | 1 | 1 | 1 | 1 | 1 |
| ever | 1.182 (.885 - 1.580) | 1.252 (.919 - 1.705) | 1.321 (.965 - 1.809) | 1.320 (.944 - 1.847) | 1.355 (.948 - 1.936) | 1.353 (.951 - 1.926) |
| **Physical violence by peers** |  |  |  |  |  |  |
| never | 1 | 1 | 1 | 1 | 1 | 1 |
| ever | 1.220 (.948 - 1.569) | 1.278 (.970 - 1.684) | 1.202 (.910 - 1.587) | 1.058 (.792 - 1.415) | 1.022 (.742 - 1.408) | 1.014 (.740 - 1.390) |
| **Psychological violence by peers** |  |  |  |  |  |  |
| never | 1 | 1 | 1 | 1 | 1 | 1 |
| ever | 1.095 (.829 - 1.445) | 1.137 (.841 - 1.538) | 1.120 (.827 - 1.515) | 0.995 (.722 - 1.371) | 1.046 (.726 - 1.506) | 1.038 (.723 - 1.492) |

Model 1 – adjusted for age, marital status and socio-economic status

Model 2 – adjusted for all variables of Model 1 + social support and number of children

Model 3 – adjusted for all variables of Model 2 + overall health status, alcohol consumption and chronic condition

Model 4 – adjusted for all variables of Model 3 + gynecologist in the last 12 months and cervix cancer smear (ever)

Model 5 – adjusted for all variables of Model 4 + diabetes and hypertension

Weighted = population weights according to RKI, 2020b

aOR= adjusted Odds Ratio, adjusted for all other variables in the model

95%CI= 95% Confidence Interval

* = not included in the final model

**Table S9. Confounders of the association between violence and current contraceptive use**

|  | **Sexual and reproductive health outcome** | | | | | |
| --- | --- | --- | --- | --- | --- | --- |
|  | **Current contraceptive use** | | | | | |
| **Violence** | **Crude Model Weighted OR (95% CI)** | **Model 1 Weighted aOR (95% CI)** | **Model 2 Weighted aOR (95% CI)** | **Model 3 Weighted aOR (95% CI)** | **Model 4 Weighted aOR (95% CI)*** | **Model 5 Weighted aOR (95% CI)*** |
| **Physical violence 12 months** |  |  |  |  |  |  |
| no | 1 | 1 | 1 | 1 | 1 | 1 |
| yes | 1.378 (.829 - 2.291) | .842 (.455 - 1.557) | .841 (.440 - 1.611) | .845 (.421 - 1.694) | 1.101 (.457 - 2.653) | 1.094 (.459 - 2.610) |
| **Psychological violence 12 months** |  |  |  |  |  |  |
| no | 1 | 1 | 1 | 1 | 1 | 1 |
| yes | **1.404 (1.109 - 1.778)** | 1.082 (.804 - 1.455) | 1.118 (.821 - 1.521) | 1.051 (.768 - 1.437) | 1.131 (.813 - 1.573) | 1.134 (.815 - 1.577) |
| **Physical violence since the age of 16** |  |  |  |  |  |  |
| no | 1 | 1 | 1 | 1 | 1 | 1 |
| yes | .995 (.741 - 1.335) | .883 (.605 - 1.289) | .879 (.590 - 1.307) | .859 (.547 - 1.351) | 1.001 (.615 - 1.631) | 1.042 (.631 - 1.719) |
| **Psychological violence since the age of 16** |  |  |  |  |  |  |
| no | 1 | 1 | 1 | 1 | 1 | 1 |
| yes | **1.280 (1.041 - 1.574)** | 1.002 (.777 - 1.292) | 1.001 (.776 - 1.290) | .992 (.756 - 1.300) | .989 (.741 - 1.320) | .993 (.744 - 1.327) |
| **Physical violence by parent/caregiver** |  |  |  |  |  |  |
| never | 1 | 1 | 1 | 1 | 1 | 1 |
| ever | **.708 (.580 - .865)** | .826 (.647 - 1.053) | .846 (.656 - 1.090) | .806 (.623 - 1.044) | .799 (.613 - 1.043) | .803 (.615 - 1.049) |
| **Psychological violence by parents/caregiver** |  |  |  |  |  |  |
| never | 1 | 1 | 1 | 1 | 1 | 1 |
| ever | 1.083 (.866 - 1.354) | 1.031 (.806 - 1.318) | 1.020 (.788 - 1.321) | .937 (.732 - 1.200) | .884 (.686 - 1.139) | .879 (.682 - 1.133) |
| **Physical violence by peers** |  |  |  |  |  |  |
| never | 1 | 1 | 1 | 1 | 1 | 1 |
| ever | .854 (.703 - 1.037) | **.702 (.562 - .876)** | **.689 (.544 - .871)** | **.644 (.505 .821)** | **.632 (.489 - .817)** | **.634 (.490 - .820)** |
| **Psychological violence by peers** |  |  |  |  |  |  |
| never | 1 | 1 | 1 | 1 | 1 | 1 |
| ever | **1.437 (1.155 - 1.786)** | 1.007 (.782 - 1.297) | .976 (.743 - 1.282) | .913 (.697 -1.197) | .927 (.706 - 1.218) | .929 (.707 - 1.221) |

Model 1 – adjusted for age, marital status and socio-economic status

Model 2 – adjusted for all variables of Model 1 + social support and number of children

Model 3 – adjusted for all variables of Model 2 + overall health status, alcohol consumption and chronic condition

Model 4 – adjusted for all variables of Model 3 + gynecologist in the last 12 months and cervix cancer smear (ever)

Model 5 – adjusted for all variables of Model 4 + diabetes and hypertension

Weighted = population weights according to RKI, 2020b

aOR= adjusted Odds Ratio, adjusted for all other variables in the model

95%CI= 95% Confidence Interval

* = not included in the final model

**Table S10. Confounders of the association between violence and miscarriage or stillbirth (ever)**

|  | **Sexual and reproductive health outcome** | | | | | |
| --- | --- | --- | --- | --- | --- | --- |
|  | **Ever miscarriage or stillbirth** | | | | | |
| **Violence** | **Crude Model Weighted OR (95% CI)** | **Model 1 Weighted aOR (95% CI)** | **Model 2 Weighted aOR (95% CI)** | **Model 3 Weighted aOR (95% CI)** | **Model 4 Weighted aOR (95% CI)*** | **Model 5 Weighted aOR (95% CI)*** |
| **Physical violence 12 months** |  |  |  |  |  |  |
| no | 1 | 1 | 1 | 1 | 1 | 1 |
| yes | .703 (.312 - 1.582) | 1.141 (.493 - 2.638) | 1.464 (.613 - 3.497) | 1.282 (.497 - 3.309) | 1.281 (.487 - 3.373) | 1.283 (.489 - 3.367) |
| **Psychological violence 12 months** |  |  |  |  |  |  |
| no | 1 | 1 | 1 | 1 | 1 | 1 |
| yes | **.597 (.430 - .829)** | .771 (.540 - 1.100) | .800 (.550 - 1.163) | .800 (.534 - 1.199) | .822 (.535 - 1.263) | .825 (.538 - 1.258) |
| **Physical violence since the age of 16** |  |  |  |  |  |  |
| no | 1 | 1 | 1 | 1 | 1 | 1 |
| yes | **1.645 (1.127 - 2.401)** | **1.657 (1.102 - 2.492)** | **1.794 (1.148 - 2.806)** | **1.887 (1.171 - 3.043)** | **1.865 (1.155 - 3.014)** | **1.918 (1.177 - 3.124)** |
| **Psychological violence since the age of 16** |  |  |  |  |  |  |
| no | 1 | 1 | 1 | 1 | 1 | 1 |
| yes | 1.134 (.852 - 1.510) | **1.495 (1.069 - 2.091)** | **1.668 (1.165 - 2.387)** | **1.562 (1.061 - 2.298)** | 1.486 (.979 - 2.256) | 1.508 (.995 - 2.285) |
| **Physical violence by parent/caregiver** |  |  |  |  |  |  |
| never | 1 | 1 | 1 | 1 | 1 | 1 |
| ever | **1.463 (1.134 - 1.888)** | 1.174 (.885 - 1.555) | 1.200 (.895 - 1.609) | 1.203 (.905 - 1.600) | 1.259 (.946 - 1.675) | 1.246 (.933 - 1.664) |
| **Psychological violence by parents/caregiver** |  |  |  |  |  |  |
| never | 1 | 1 | 1 | 1 | 1 | 1 |
| ever | 1.188 (.905 - 1.561) | 1.241 (.932 - 1.654) | 1.316 (.985 - 1.759) | **1.387 (1.023 - 1.881)** | 1.343 (.978 - 1.843) | 1.328 (.966 - 1.827) |
| **Physical violence by peers** |  |  |  |  |  |  |
| never | 1 | 1 | 1 | 1 | 1 | 1 |
| ever | 1.184 (.892 - 1.571) | 1.217 (.904 - 1.639) | 1.237 (.917 - 1.669) | 1.170 (.855 - 1.601) | 1.233 (.901 - 1.688) | 1.231 (.896 - 1.692) |
| **Psychological violence by peers** |  |  |  |  |  |  |
| never | 1 | 1 | 1 | 1 | 1 | 1 |
| ever | .864 (.671 - 1.113) | 1.130 (.855 - 1.494) | 1.149 (.864 - 1.529) | 1.187 (.870 - 1.620) | 1.169 (.849 - 1.609) | 1.162 (.842 - 1.603) |

Model 1 – adjusted for age, marital status and socio-economic status

Model 2 – adjusted for all variables of Model 1 + social support and number of children

Model 3 – adjusted for all variables of Model 2 + overall health status, alcohol consumption and chronic condition

Model 4 – adjusted for all variables of Model 3 + gynecologist in the last 12 months and cervix cancer smear (ever)

Model 5 – adjusted for all variables of Model 4 + diabetes and hypertension

Weighted = population weights according to RKI, 2020b

aOR= adjusted Odds Ratio, adjusted for all other variables in the model

95%CI= 95% Confidence Interval

* = not included in the final model

**Table S11. Confounders of the association between violence and abortion (ever)**

|  | **Sexual and reproductive health outcome** | | | | | |
| --- | --- | --- | --- | --- | --- | --- |
|  | **Ever abortion** | | | | | |
| **Violence** | **Crude Model Weighted OR (95% CI)** | **Model 1 Weighted aOR (95% CI)** | **Model 2 Weighted aOR (95% CI)** | **Model 3 Weighted aOR (95% CI)** | **Model 4 Weighted aOR (95% CI)*** | **Model 5 Weighted aOR (95% CI)*** |
| **Physical violence 12 months** |  |  |  |  |  |  |
| no | 1 | 1 | 1 | 1 | 1 | 1 |
| yes | .930 (.418 - 2.067) | 1.315 (.574 - 3.014) | 1.293 (.510 - 3.279) | 1.481 (.581 - 3.773) | 1.746 (.647 - 4.711) | 1.692 (.619 - 4.630) |
| **Psychological violence 12 months** |  |  |  |  |  |  |
| no | 1 | 1 | 1 | 1 | 1 | 1 |
| yes | .827 (.591 - 1.158) | 1.025 (.697 - 1.507) | 1.041 (.685 - 1.580) | 1.029 (.676 - 1.628) | 1.036 (.652 - 1.647) | 1.026 (.644 - 1.634) |
| **Physical violence since the age of 16** |  |  |  |  |  |  |
| no | 1 | 1 | 1 | 1 | 1 | 1 |
| yes | **1.826 (1.230 - 2.711)** | **1.887 (1.233 - 2.888)** | **1.685 (1.014 - 2.800)** | 1.608 (.945 - 2.735) | 1.561 (.905 - 2.691) | 1.541 (.890 - 2.668) |
| **Psychological violence since the age of 16** |  |  |  |  |  |  |
| no | 1 | 1 | 1 | 1 | 1 | 1 |
| yes | 1.128 (.825 - 1.543) | 1.387 (.984 - 1.956) | 1.520 (1.047 - 2.206) | 1.446 (.983 - 2.129) | **1.516 (1.023 - 2.248)** | **1.490 (1.006 - 2.208)** |
| **Physical violence by parent/caregiver** |  |  |  |  |  |  |
| never | 1 | 1 | 1 | 1 | 1 | 1 |
| ever | **1.442 (1.071 - 1.941)** | 1.163 (.861 - 1.572) | 1.132 (.827 - 1.550) | 1.086 (.789 - 1.495) | 1.066 (.764 - 1.488) | 1.070 (.767 - 1.494) |
| **Psychological violence by parents/caregiver** |  |  |  |  |  |  |
| never | 1 | 1 | 1 | 1 | 1 | 1 |
| ever | **1.755 (1.291 - 2.385)** | **1.831 (1.315 - 2.549)** | **1.868 (1.313 - 2.659)** | **1.874 (1.299 - 2.702)** | **1.846 (1.271 - 2.682)** | **1.832 (1.262 - 2.659)** |
| **Physical violence by peers** |  |  |  |  |  |  |
| never | 1 | 1 | 1 | 1 | 1 | 1 |
| ever | .972 (.726 - 1.301) | 1.010 (.751 - 1.359) | .993 (.732 - 1.345) | .910 (.662 - 1.251) | .909 (.653 - 1.265) | .908 (.652 - 1.264) |
| **Psychological violence by peers** |  |  |  |  |  |  |
| never | 1 | 1 | 1 | 1 | 1 | 1 |
| ever | 1.022 (.761 - 1.372) | 1.336 (.959 - 1.860) | 1.417 (.990 - 2.029) | 1.395 (.968 - 2.010) | **1.483 (1.034 - 2.126)** | **1.477 (1.030 - 2.119)** |

Model 1 – adjusted for age, marital status and socio-economic status

Model 2 – adjusted for all variables of Model 1 + social support and number of children

Model 3 – adjusted for all variables of Model 2 + overall health status, alcohol consumption and chronic condition

Model 4 – adjusted for all variables of Model 3 + gynecologist in the last 12 months and cervix cancer smear (ever)

Model 5 – adjusted for all variables of Model 4 + diabetes and hypertension

Weighted = population weights according to RKI, 2020b

aOR= adjusted Odds Ratio, adjusted for all other variables in the model

95%CI= 95% Confidence Interval

* = not included in the final model

**Table S12. Comparison between participants with full valid information and those with at least one missing value in one of the violence variables analysed.**

| Socio-demographic characteristics | | Full info  n unweighted (% weighted) | At least one missing  n unweighted (% weighted) | p-value (χ²) |
| --- | --- | --- | --- | --- |
| **Age** | |  |  |  |
|  | 18-24 years | 297 (13.4) | 15 (8.2) | 0.004 |
|  | 25-34 years | 455 (19.5) | 36 (14.1) |  |
|  | 35-44 years | 611 (22.6) | 65 (19.8) |  |
|  | 45-54 years | 808 (25.3) | 97 (29.3) |  |
|  | 55-64 years | 662 (19.2) | 103 (28.6) |  |
| **Socioeconomic Status** | |  |  |  |
|  | low | 370 (16.8) | 57 (28.2) | 0.001 |
|  | medium | 1769 (62.9) | 171 (56.3) |  |
|  | high | 684 (20.3) | 59 (15.6) |  |

Significance is based on the adjusted F and its degrees of freedom. The adjusted F is a variant of the second-order Rao-Scott adjusted chi-square statistic.
