# Supplementary material for: Sexual and reproductive health outcomes of women who experienced violence in Germany: Analysis of the German health interview and examination survey for adults (DEGS1)
Source: PLoS One. 2024 Aug 9;19(8):e0305992. doi: 10.1371/journal.pone.0305992 (PMC11315302; doi:10.1371/journal.pone.0305992)
Supplement: Attachment — Submitted filename: Response to Reviewers.docx [file pone.0305992.s002.docx]

Dear Editor,

Thank you for the opportunity to revise our manuscript: “Violence against women in adulthood and childhood, and sexual and reproductive health outcomes: analysis of the German Health Interview and Examination Survey for Adults (DEGS1)”.

Please find below a point-by-point response to the reviewers concerns and find enclosed the revised version of the manuscript, where amendments are highlighted using tracked changes. A clean version is also enclosed.

We believe the changes proposed by the reviewers significantly improved the quality of our manuscript and we thank them.

Data Availability statement:

The data that support the findings of this study are owned by a third-party organization. The data are available from the Robert Koch Institute (available as Scientific Use File) but restrictions apply to the availability of these data, which were used under license for the current study, and so are not publicly available. Further information including instructions on how to request a Scientific Use File of these data can be found in the RKI website (https://www.rki.de/DE/Content/Forsch/FDZ/Zugang/SUF.html).

We hope this version complies with the standards of PLOS ONE for publication.

Yours sincerely,

Diogo Costa

Department of Population Medicine and Health Services Research,

School of Public Health, Bielefeld University,

P.o. Box 10 01 31, D- 33501 Bielefeld, Germany

**Additional Editor Comments:**

*In addition to the reviewer, Could you please include more elaboration for your 'multivariate" logistic regression ? Is that really a multi-variate analysis ?*

**Response**: We thank the Editor for this remark, and we now refer to our models as multivariable. Our models are multivariable or multiple logistic regression models because each model has one outcome variable and multiple predictors.

We changed “multivariate” for “multivariable” in the abstract (page 2, line 27 of the revised manuscript), methods (page 11, lines 251 and detailed in lines 260-261), and results sections (page 19, line 381).

**Reviewers' comments:**

**Reviewer #1:**

**1.** *Title*

*Change the title to*

*The sexual and reproductive health outcomes of women who experienced violence in adulthood and childhood in Germany.*

**Response:** We thank the reviewer suggestion and followed the recommendation for both the full and short titles. We still would like to keep the statement regarding the analysis conducted in the scope of the German Health Interview and Examination Survey for Adults. We, therefore, changed the full title to:

“Sexual and reproductive health outcomes of women who experience violence in adulthood and childhood in Germany: analysis of the German Health Interview and Examination Survey for Adults (DEGS1)”, and the short title to: “Sexual and reproductive health outcomes of women who experienced violence in Germany”.

**2.** *Abstract*

*Remove all the words abbreviation in the study abstract except “aOR”*

**Response:** We removed SRH and DEGS1 abbreviations from the abstract and kept “aOR”.

**3*.*** *Line 16 to 17 should be rewritten as “ this study examined the association between women exposure to physical violence, psychological violence and sexual & reproductive health outcomes………..*

**Response:** We rephrased line 17 as suggested – “this study examined the association between women exposure to physical violence, psychological violence and sexual and reproductive health outcomes”, (lines 19-21 of the revised manuscript).

**4.** *Collapse the study design under methods. Then the method section should read as “**This study used a cross-sectional research design to collect data on violence……….*

**Response:** We inserted the study design under methods as suggested – “This study used a cross-sectional research design to collect data on violence…” (line 24).

**5.** *I am not too comfortable with the use of this repeated word “violence against” instead use the word “women who experienced violence….”*

**Response:** The term violence against women corresponds to the terminology used by the World Health Organisation, the United Nations, the Council of Europe, among several other organizations, and is embedded in international and regional legislation and relevant policy documents. We, therefore, changed the term as suggested in several places throughout the manuscript, but also kept it in parts of the text that refer to other studies or reports where it is used.

**6.** *The level of significant should be stated in the abstract*

**Response:** Significance level was added to the abstract (p<0.05), line 32.

**7.** *Change “confidence interval” to CI*

**Response:** Changed accordingly.

**8.** *Change “negative” to adverse.*

**Response**: Changed accordingly, line 39.

**9.** *Introduction*

*Wrong journal reference style. Use PLoS recommended reference formatting.*

**Response**: The manuscript is now formatted using PLOS style.

**10.** *More information is needed in the introduction section on how IPV relates to other SRH variables included in this study. More emphasizes was made on contraceptives neglecting other variables. At the same times it’s important to give some fact on other regional prevalence and the need for this study as a global health issue*

**Response***:* Two paragraphs were added to the introduction section, emphasising the associations between IPV and other SRH outcomes, also considering this problem as a global issue, resorting to a recently published longitudinal study and a systematic review. The specific text and references added were (page 4, lines 74-85):

“In a longitudinal analysis conducted in Australia during 2021-2022 among two cohorts, IPV exposure was shown associated with increased odds of experiencing several sexual and reproductive health outcomes, including infertility, termination and miscarriage, which showed a dose-response effect, i.e., increased odds of experience adverse outcomes with greater exposure to IPV [1].

A recent systematic review on IPV and perinatal health included 15 studies conducted in Asia, 12 in North America and Oceania, 12 in Central and South America, 6 in Africa and 5 in Europe, to study the relationship between IPV and 39 different outcomes [2]. Amongst the studies classified with high methodological quality, ten studies included miscarriage as an outcome and six of them found an association with IPV (with miscarriage risks ranging between 1.7 and 5.7 times higher among women experiencing IPV). In the same review, among seven studies with high methodological quality that reported outcomes of perinatal death, six studies associated IPV experiences with this outcome (with risk ranging between 1.4 and 6.04 times higher)”.

References:

1. Hutchinson M, Cosh SM, East L. Reproductive and sexual health effects of intimate partner violence: A longitudinal and intergenerational analysis. Sex Reprod Healthc. 2023;35: 100816. doi:10.1016/j.srhc.2023.100816

2. Pastor‐Moreno G, Ruiz‐Pérez I, Henares‐Montiel J, Escribà‐Agüir V, Higueras‐Callejón C, Ricci‐Cabello I. Intimate partner violence and perinatal health: a systematic review. BJOG An Int J Obstet Gynaecol. 2020;127: 537–547. doi:10.1111/1471-0528.16084

**11.** *Lastly in the last paragraph please tell us how the study will contribute to global public health needs in relation to SRH*

**Response**: The following sentence was added to the last paragraph of the introduction, emphasizing the need to address IPV and its association with adverse SRH as a global public health issue, for which the current contextualized results can contribute (page 5, lines 104-107):

“These contextualized results can help raise awareness about the adverse effects that violence experiences have in SRH outcomes, and about the need to address this global public health issue through a multisectoral approach that aims at violence prevention”.

**12**. *Methods*

*I have so much reservations on the operationalization of the outcome variables*

*This study is on SRH outcome and beware that IPV is also part of SRH dimensions (This means there could some elements of collinearity) – You must provide the collinearity test*

**Response**: In line with the studies cited and the more recent references added, our objective is to measure the magnitude of the association between IPV and SRH and treating different forms of IPV as single determinants of separate adverse SRH outcomes. The fact that IPV may be part of a dimension of SRH shows its pervasiveness and the need to prevent it.

Since we stratified our analysis according to each type of violence assessed and each SRH outcome, we did not assess multicollinearity among these variables - models were fitted separately for each SRH outcome and each type of violence assessed. Therefore, Table 5 in the manuscript shows the results of the 32 separate final adjusted models (8 violence types X 4 SRH outcomes). This clarification was added to the methods section, page 12, lines 279-285.

**13.** *The SRH is measure between age 15-49 covering the fecundity period and also to ensure better analytical output of unskewed dataset – Dataset must be restricted to this age group*

**Response**: We thank the reviewer observation and acknowledge the normal period when these outcomes are assessed. However, we treated violence experiences as the dependent variable in our study and analysed the lifetime report of each SRH. Our interest was to look into women who had ever experienced, for example a miscarriage or stillbirth or abortion, in their lifetime. Acknowledging the limitations of the cross-sectional analysis to infer about the direction of the associations, we investigated the distribution of each SRH outcome according to the demographics characteristics of participants and noted that all of them were observed across all the age groups analysed (18-64 years), as shown in Supplementary Tables S6 and S7. The only characteristic analysed for which the questioning referred to women’s current situation (i.e. at the time of interviewing), was “current contraceptive use”, which could be hypothesised to be present only during the fecundity period. However, we noted that current contraceptive use was reported by 255 women in the age group 45-54 years old and by 14 women in the age group 55-64 years old (Supplementary Table S6). We, therefore, included all women in the analysis, and adjusted our models for age to address the potential confounding from the age distribution of this characteristic. We added this explanation to the Strengths and limitations section of the manuscript (page 29, lines 599-603).

**14.** *Birth control pill is a form of modern contraceptive which could have been added to those using contraceptive – how are you able to unmerge these participants*

**Response**: We thank the reviewer observation and acknowledge this as a limitation in our study. The questionnaire asked if women ever used birth control pill and current contraceptive, using two different questions, which we analysed as separate characteristics. Therefore, we were not able to test the effect of violence experiences over the accumulation of contraceptive methods. This was added to the Strengths and limitations section of the manuscript (page 29, lines 582-584).

**15.** *Include how to access the dataset and permission needed*

**Response**: The following data availability statement was shared:

The data that support the findings of this study are owned by a third-party organization. The data are available from the Robert Koch Institute (available as Scientific Use File) but restrictions apply to the availability of these data, which were used under license for the current study, and so are not publicly available. Further information including instructions on how to request a Scientific Use File of these data can be found in the RKI website (https://www.rki.de/DE/Content/Forsch/FDZ/Zugang/SUF.html).

**16**. *Results*

*There is a need to stick with weighted and delete all unweighted information*

**Response**: Accordingly, we deleted the columns correspondent to unweighted frequencies from Tables 1, 2 and 3.

**17.** *Missing values must be dropped because there is no way this can be useful to your results or literature needs*

**Response**: We removed lines with missing values from Tables 1, 2 and 3.

**18.** *Can we have a graph displaying the weighted percentage of all the outcome variables?*

**Response**: Two graphs were added to the results section of the manuscript:

Figure 1. Violence experiences (%, weighted), page 15.

Figure 2. Sexual and reproductive health outcomes (%, weighted), page 16.

**19**. *Discussion*

*- There are a lot of acronyms here that has not been defined previously e.g AUDIT C or CASMIN*

**Response**: We checked all acronyms and added full names where missing. AUDIT C - Alcohol Use Disorders Identification Test-Consumption, was presented in the methods sections (page 9, lines 187-188). CASMIN was removed since it was not relevant in the analysis. We thank the reviewer for these corrections.

**20.** *Change goal to objective*

**Response**: Changed accordingly (page 24, line 452).

**21.** *For quantitative study we don’t explore we examine, assess etc*

**Response**: We changed “explore” to “assess”, page 24, line 452.

**22.** *Write the summary of your result immediately after the objective*

**Response**: a short summary of the main results was added after the objectives, page 24, lines 455-458. The text added was as follows:

“We found that exposure to physical violence by a parent or caregiver increased the odds of lifetime birth control pill utilization, a miscarriage or stillbirth was more likely among women reporting exposure to physical violence since the age of 16, and an abortion was more likely among women reporting exposure to psychological violence by a parent or caregiver.”

**23.** *Remove all the “ (95%CI= 2.6 – 4.2%, weighted estimates)” make your discussion using “likelihood” “higher odds” “less likely” etc*

**Response**: We removed mentions to confidence intervals of estimates and used qualifiers as suggested throughout the discussion section.

**24.** *Compare your study to studies conducted in Africa where these issues are arguably high*

**Response**: The following paragraph and references were added to the discussion section to highlight this point (page 28, lines 559-565):

“The frequency and severity of the adverse effects of violence experiences on SRH are higher in other world regions, namely Africa, Asia and Latina America, compared to Europe, where fewer studies exist [1]. This is a reflection of the higher prevalence of IPV observed (both lifetime and during the past-year) in these regions, as shown by the latest global estimates [54], of the different levels of access and adequacy of sexual and reproductive health services (particularly evident, for example, in the sub-Saharan region [2]), and also of different levels of women´s empowerment and access to education [3].”

References:

1. Pastor‐Moreno G, Ruiz‐Pérez I, Henares‐Montiel J, Escribà‐Agüir V, Higueras‐Callejón C, Ricci‐Cabello I. Intimate partner violence and perinatal health: a systematic review. BJOG An Int J Obstet Gynaecol. 2020;127: 537–547. doi:10.1111/1471-0528.16084

2. Rosário EVN, Gomes MC, Brito M, Costa D. Determinants of maternal health care and birth outcome in the Dande Health and Demographic Surveillance System area, Angola. PLoS One. 2019;14. doi:10.1371/journal.pone.0221280

3. Garcia-Moreno C, Amin A. Violence against women: where are we 25 years after ICPD and where do we need to go? Sex Reprod Heal matters. 2019;27: 1–3. doi:10.1080/26410397.2019.1676533

**25***. Other comments*

*The strengths and limitation section is too long – concise it*

**Response**: The strengths and limitations section was shortened following the reviewer suggestion.

**26.** *Remove references*

**Response**: We would prefer not to remove the references used, as this is not a restriction listed in the journal's requirements.

**27.** *I didn’t see any conclusion and recommendation section*

**Response**: We added this title to the last section, that was meant to conclude and recommend on further actions.

**28.** *After strengths and limitations section before the conclusions and recommendations section, write a section called “implication for public health research and policy” here discuss the implications of the findings from this study to public health research and policy*

**Response**: The following paragraph was added after the Strengths and limitations section and before the Conclusions and recommendations, suggesting the implications for public health research and policy (page 30, lines 625-632):

“These results have implications for public health research and policy. In Europe, and particularly in Germany, there may be less interest in the adverse impact that violence experiences have on SRH outcomes, because of the comparatively high level of development and access to sexual and reproductive specialized services. However, our results suggest that such harsh effects are noticeable when assessing women from the German general population, particularly when considering specific forms of violence experienced during women´s lifetime (e.g., physical and psychological violence since the age of 16). This also suggests the need to include violence assessment in clinical encounters, in particular for health services dedicated to women´s health”.
