# Supplementary material for: Sexual and reproductive health outcomes of women who experienced violence in Germany: Analysis of the German health interview and examination survey for adults (DEGS1)
Source: PLoS One. 2024 Aug 9;19(8):e0305992. doi: 10.1371/journal.pone.0305992 (PMC11315302; doi:10.1371/journal.pone.0305992)
Supplement: Attachment — Submitted filename: Response AP Review.docx [file pone.0305992.s003.docx]

Response AP Review

**Abstract**

Line 21: and SRH outcomes- SRH appears for the first time as an abbreviation without it written in full anywhere else (above).

Line 23: regression models were fitted to: how about ‘used’- fitting could be more relevant when describing data depicted in a plot.

This sentence from your conclusion ‘Exposure to violence was associated with adverse sexual and reproductive health outcomes among adult German women’, supported by the stats would make your results more tangiable/fully understood or ‘have more weight’. Otherwise it’s just a ‘too technical presentation’.

**Conclusion:**

The authors wrote: “Scoping for violence experiences in clinical encounters should be considered for the prevention of women’s adverse health outcomes.”

Scoping means ‘act or practice of eyeing or examining’ -According to websearch for example:

Considered by who- make it clear?

Clinical encounters are generally by the health care provider – so they don’t do it?

Hunzelar C, Krumpholtz Y, Schlack R, Weltermann B. More GP Consultations by Violence Victims: Results from the Representative German DEGS1 Study. Int J Environ Res Public Health. 2023 Mar 6;20(5):4646. doi: 10.3390/ijerph20054646. PMID: 36901654; PMCID: PMC10001473.

This study above on the DEGS1 Germany -2008-2011 reports on those experiencing violence n=3149 (females) … they reported that those who are victims of violence consulted quite often…but in your conclusion you indicate that the health practitioners should consider it. As a reader, this is the main conclusion drawn from your study and its somehow unclear.

This is the main conclusion of the study in discussion

The high frequency of GP contacts in VE **victims constitutes opportunities to professionally support this vulnerable patient group and underlines the necessity for GPs to integrate VE as a bio-psycho-social problem in a holistic treatment approach.**

Since they analysed

n=3149 (females)- DEGS1 Germany -2008-2011 (Just like the authors)

& from my understanding came to the same conclusion as in this manuscript regarding ‘clinical encounters’

How is this study different from the one already published?

Its clear you wish to highlight more on SRH make it more visible in your conclusions!

Where in the text is the obstetrics and gynaecologist for example, organisations dealing with violence…

Hunzelar C, Krumpholtz Y, Schlack R, Weltermann B. More GP Consultations by Violence Victims: Results from the Representative German DEGS1 Study. Int J Environ Res Public Health. 2023 Mar 6;20(5):4646. doi: 10.3390/ijerph20054646. PMID: 36901654; PMCID: PMC10001473.

Interestingly, the study above made an analysis of the same database and **nowhere** does it appear on this manuscript.

The conclusion part could target the results better especially that they ‘associations statistically significant’ where, the results variables /keywords and:

Age of victim (young therefore still attending school most likely-)

Parent

caregivers

You want your research to make your research have a positive impact on health, right? Why put a recommendation towards a health care provider only? Advocate more on awareness of violence in e.g schools, now that you found out on the ‘parents’ contribution to the SRH adverse effects what do you recommend?

This is part of your manuscript conclusion:

Violence against women as well as SRH need to be recognized and treated as public health concerns and addressed through a multisectoral approach by researchers, policy makers and the healthcare sector…

**consider such statements for your abstract conclusion**

**Background (no comments)**

**Methodology**

Line 114: such as residential homes [33,34] (n=8,152).

Consider position of ((n=8,152) … ? before references

Line 124….4,193: Rules on starting sentences with numbers (remember them)

**Results:**

‘German citizens, people of foreign nationality and people living in institutions

such as residential homes’

The researcher anticipates a difference in outcomes between these population groups., influenced by SES, Origin, migration status among foreign national etc…

Any rationale for not doing a subgroup analysis or test of association between the variables above with SRH outcomes.

Line 547 – 553 of your discussion

The frequency and severity of the adverse effects of violence experiences on SRH are higher in other world regions, namely Africa, Asia and Latina America, compared to Europe, where fewer studies exist [20]….

Don’t you think it worth to check outcomes of the population from these regions now living in Europe in your study?

Which nation experienced the worst outcomes? Or did the reviewer miss it, its somewhere? / rationale for not doing the analysis.

Ps: This helps in designing the appropriate / relevant Interventions/ recommendations to combat all negative outcome.

**Discussion:**

Line 462: survey designs as FRA and BMFSFJ: check that the 2 abbreviations were written in full somewhere before discussion.

Line 576: two full stops-typo?

**Reference:**

Numerous mistakes that are scientifically not OK! Please check every reference

Examples:

Reference 1-9 , 31, 38, 39…
