# Supplementary material for: Sexual and reproductive health outcomes of women who experienced violence in Germany: Analysis of the German health interview and examination survey for adults (DEGS1)
Source: PLoS One. 2024 Aug 9;19(8):e0305992. doi: 10.1371/journal.pone.0305992 (PMC11315302; doi:10.1371/journal.pone.0305992)
Supplement: Attachment — Submitted filename: Response to Reviewers_03june2024.docx [file pone.0305992.s004.docx]

Dear Editor,

Thank you for the opportunity to revise our manuscript: “Sexual and reproductive health outcomes of women who experienced violence in adulthood and childhood in Germany: analysis of the German Health Interview and Examination Survey for Adults (DEGS1)”.

Please find below a point-by-point response to the academic editor and reviewers concerns and find enclosed the marked-up and unmarked versions of the revised manuscript.

We believe the changes proposed by the reviewers significantly improved the quality of our manuscript and we thank them.

We hope this version complies with the standards of PLOS ONE for publication.

Yours sincerely,

Diogo Costa

Department of Population Medicine and Health Services Research, School of Public Health, Bielefeld University, Bielefeld, Germany & Research Centre for Human Development (CEDH), Faculty of Education and Psychology, Universidade Católica Portuguesa, Porto, Portugal

**Academic Editor**

*In my reading, there are clearly other studies/manuscripts that have been written using the same data sources you have used but you have not considered these in your manuscript. These manuscripts will be helpful in contextualising your results and situating them along what others have found. See for example; Hunzelar C, Krumpholtz Y, Schlack R, Weltermann B. More GP Consultations by Violence Victims: Results from the Representative German DEGS1 Study. Int J Environ Res Public Health. 2023 Mar 6;20(5):4646. doi: 10.3390/ijerph20054646. PMID: 36901654; PMCID: PMC10001473.*

**Response:** We have added this study as a reference (ref #31) and a brief sentence about its main results to the introduction section of our revised manuscript (page 6, lines 103-109). Please note that we submitted our manuscript to PLOS ONE in October 2022, when this article had not yet been published.

**Review Comments to the Author**

*I like the author’s integration of Sexual and reproductive health outcomes and violence. I am not sure whether it's necessary to include “adulthood and childhood” in the title. The author could leave the two concepts out of the title and highlight them in the work, explaining them further in the methodology. Explain a bit more about what you mean by multivariate logistic regression.*

**Response:** We thank the reviewer comment and removed the terms “adulthood and childhood” from the title, while clarifying how these were defined in the methods section (page 9, line 177).

In the previous round of reviews, we corrected the term multivariate for multivariable, as this was what we did: our models are multivariable or multiple logistic regression models because each model has one outcome variable and multiple predictors. This was added to the methods section (page 12, lines 269-270).

**AP Review**

**Abstract**

***1.*** *Line 21: and SRH outcomes- SRH appears for the first time as an abbreviation without it written in full anywhere else (above).*

**Response**: We thank the reviewer for the careful reading of our manuscript and suggestions. We revised accordingly, page 2, line 23 – Sexual and reproductive health (SRH).

***2.*** *Line 23: regression models were fitted to: how about ‘used’- fitting could be more relevant when describing data depicted in a plot.*

**Response**: Changed accordingly throughout the manuscript (page 2 line 25, page 12 line 260, and page 13 line 280).

***3.*** *This sentence from your conclusion ‘Exposure to violence was associated with adverse sexual and reproductive health outcomes among adult German women’, supported by the stats would make your results more tangiable/fully understood or ‘have more weight’. Otherwise it’s just a ‘too technical presentation’.*

**Response:** We thank the reviewer suggestion and changed the sentence as follows (page 2, lines 35-37): “The results suggest that adult German women who experienced physical or psychological violence since the age of 16, including violence perpetrated by a parent or caregiver, were more likely to report miscarriage or stillbirth and abortion”.

***4. Conclusion:***

*The authors wrote: “Scoping for violence experiences in clinical encounters should be considered for the prevention of women’s adverse health outcomes.”*

*Scoping means ‘act or practice of eyeing or examining’ -According to websearch for example:*

*Considered by who- make it clear?*

*Clinical encounters are generally by the health care provider – so they don’t do it?*

**Response**: We rephrased the sentence in the abstract to make it clearer and to answer the specific questions raised by the reviewer. The sentence now reads as follows (page 2, lines 37-43): “Direct assessment of violence experiences against women should be conducted by healthcare professionals in clinical encounters, particularly by obstetrics and gynaecological specialists, for the prevention of women´s adverse sexual and reproductive health outcomes. Furthermore, violence should be treated as a major public health concern and addressed through a multisectoral approach, involving the healthcare and educational sectors, researchers and relevant policymakers”.

***5****. Hunzelar C, Krumpholtz Y, Schlack R, Weltermann B. More GP Consultations by Violence Victims: Results from the Representative German DEGS1 Study. Int J Environ Res Public Health. 2023 Mar 6;20(5):4646. doi: 10.3390/ijerph20054646. PMID: 36901654; PMCID: PMC10001473.*

*This study above on the DEGS1 Germany -2008-2011 reports on those experiencing violence n=3149 (females) … they reported that those who are victims of violence consulted quite often…but in your conclusion you indicate that the health practitioners should consider it. As a reader, this is the main conclusion drawn from your study and its somehow unclear.*

*This is the main conclusion of the study in discussion*

*The high frequency of GP contacts in VE* ***victims constitutes opportunities to professionally support this vulnerable patient group and underlines the necessity for GPs to integrate VE as a bio-psycho-social problem in a holistic treatment approach.***

*Since they analysed n=3149 (females)- DEGS1 Germany -2008-2011 (Just like the authors) & from my understanding came to the same conclusion as in this manuscript regarding ‘clinical encounters’ How is this study different from the one already published?*

*Its clear you wish to highlight more on SRH make it more visible in your conclusions!*

*Where in the text is the obstetrics and gynaecologist for example, organisations dealing with violence…*

**Response**: We thank the reviewer’s remark and made changes accordingly. Specifically, we introduced the main result of the study by Hunzelar et al., (2023) in the introduction section of our revised manuscript (page 6 lines 102-105), to state that despite their findings, the study did not explore the relationship between women who experienced violence and sexual and reproductive health outcomes. We added a reference to the study.

We also changed our conclusions in the abstract to more clearly reflect our findings, as suggested (page 2, lines 35-43): “The results suggest that adult German women who experienced physical or psychological violence since the age of 16, including violence perpetrated by a parent or caregiver, were more likely to report miscarriage or stillbirth and abortion. Direct assessment of violence experiences against women should be conducted by healthcare professionals in clinical encounters, particularly by obstetrics and gynaecological specialists, for the prevention of women´s adverse sexual and reproductive health outcomes. Furthermore, violence should be treated as a major public health concern and addressed through a multisectoral approach, involving the healthcare and educational sectors, researchers and relevant policymakers.”

***6****. Hunzelar C, Krumpholtz Y, Schlack R, Weltermann B. More GP Consultations by Violence Victims: Results from the Representative German DEGS1 Study. Int J Environ Res Public Health. 2023 Mar 6;20(5):4646. doi: 10.3390/ijerph20054646. PMID: 36901654; PMCID: PMC10001473.*

*Interestingly, the study above made an analysis of the same database and* ***nowhere*** *does it appear on this manuscript.*

**Response**: Indeed. This is because the cited study was not published when we submitted our manuscript to PLOS ONE. We have now integrated its findings in our introduction section (page 6, lines 102-105) and added the reference (ref #31).

***7****. The conclusion part could target the results better especially that they ‘associations statistically significant’ where, the results variables /keywords and: Age of victim (young therefore still attending school most likely-)*

*Parent*

*caregivers*

*You want your research to make your research have a positive impact on health, right? Why put a recommendation towards a health care provider only? Advocate more on awareness of violence in e.g schools, now that you found out on the ‘parents’ contribution to the SRH adverse effects what do you recommend?*

**Response**: We have now rephrased the conclusions to reflect the importance of these topics. Specifically, we added that our results suggest that “adult German women who experienced physical or psychological violence since the age of 16, including violence perpetrated by a parent or caregiver, were more likely to report miscarriage or stillbirth and abortion” (page 2, lines 35-37) and that “violence should be treated as a major public health concern and addressed through a multisectoral approach, involving the healthcare and educational sectors, researchers and relevant policymakers” (page 2-3, lines 41-43).

***8****. This is part of your manuscript conclusion:*

*Violence against women as well as SRH need to be recognized and treated as public health concerns and addressed through a multisectoral approach by researchers, policy makers and the healthcare sector…*

***consider such statements for your abstract conclusion***

**Response:** Thank you. We have added such elements as mentioned above (pages 2-3, lines 35-43).

***9. Methodology***

*Line 114: such as residential homes [33,34] (n=8,152).*

*Consider position of ((n=8,152) … ? before references*

**Response**: Relocated to line 119, thank you for spotting this.

***10****. Line 124….4,193: Rules on starting sentences with numbers (remember them)*

**Response**: Changed the start of the sentence (line 131: “Of these, 4.193 were first-time participants”).

***11. Results:***

*‘German citizens, people of foreign nationality and people living in institutions*

*such as residential homes’*

*The researcher anticipates a difference in outcomes between these population groups., influenced by SES, Origin, migration status among foreign national etc…*

*Any rationale for not doing a subgroup analysis or test of association between the variables above with SRH outcomes.*

**Response**: The mentioned categories were used as inclusion criteria for participants when implementing the DEGS survey and were not assessed specifically for their potential relationship with different outcomes. Still, we included in our analysis several potential confounding factors (e.g. socioeconomic status, marital status, social support). However, we cannot rule out the potential for residual confounding by unmeasured variables, which is common to all observational studies. We added this limitation to our discussion section (page 30, lines 603-604).

***12****. Line 547 – 553 of your discussion*

*The frequency and severity of the adverse effects of violence experiences on SRH are higher in other world regions, namely Africa, Asia and Latina America, compared to Europe, where fewer studies exist [20]….*

*Don’t you think it worth to check outcomes of the population from these regions now living in Europe in your study?*

*Which nation experienced the worst outcomes? Or did the reviewer miss it, its somewhere? / rationale for not doing the analysis.*

*Ps: This helps in designing the appropriate / relevant Interventions/ recommendations to combat all negative outcome.*

**Response**: The reviewer raises a very pertinent point. However, we did not access or analyzed variables related to participants country of origin. Still, we think this should be done in the future and added such statement to our discussion section (page 29, lines 569-571): “Future studies conducted in Europe should consider analyzing the relationships between violence experiences and SRH within women from specific regions or countries, which could potentially help disentangle relevant influences and design concrete recommendations”.

***13. Discussion:***

*Line 462: survey designs as FRA and BMFSFJ: check that the 2 abbreviations were written in full somewhere before discussion.*

**Response**: Added the full names accordingly, page 25, lines 473 and 476.

***14****. Line 576: two full stops-typo?*

**Response**: Corrected, thank you.

***15. Reference:***

*Numerous mistakes that are scientifically not OK! Please check every reference*

*Examples:*

*Reference 1-9 , 31, 38, 39…*

**Response**: Thank you for the careful reading. We have now revised all references.
